# Montreal Veterinary Medical Association

**Published:** 1894-01

**Authors:** 


### MONTREAL VETERINARY MEDICAL ASSOCIATION.

A meeting of the Montreal Veterinary Medical Association was held in the lecture room of the Veterinary College, 6 Union Avenue, Thursday evening, Dec. 14th, the Hon. president occupying the chair for the evening.

After the reading of the minutes of the previous meeting, and transaction of regular business, Mr. A. H. Hall reported a case—operation on the pig—which was successfully performed, and good results obtained. It also brought out considerable discussion from members present.*

Mr. J. R Hollingsworth read an essay on "The external Parasites of the Horse," describing the different species of the various parasites, vegetable and animal, their mode of living under their varying circumstances, such as occupying different parts of the animal body, their manner of propagation, tenacity of life, and their effects on the animal system, producing many forms of disease, external and internal, which in many cases are contagious to man and beast.

Prof. D. McEachran in his remarks stated that the chief difficulty in treating contagious diseases produced by these parasites was due largely to lack of attention and proper regard for cleanliness of surroundings. He also gave treatment in detail by which these troublesome diseases might be eradicated.†

The next essayist, Mr. J. McLeod, presented an elaborate paper, the subject being "Pleuro-pneumonia Contagiosa in Cattle." Firstly he discussed the early history of this highly contagious disease in the bovine species as early as 350 years B. C., also referring to the various outbreaks occurring within the past 180 years in Great Britian and the United States, and the enormous loss of stock and capital invested, and the great expense to the different countries in trying to suppress these epidemics. The origin of this disease is not definitely known. The period of incubation is indefinite, the diagnosis not always certain. The symptoms were described minutely and the general appearance and course of the disease, showing variations that take place in the various stages while running its course, as observed by methods at the disposal of the veterinary practitioner. He also gave an account of rules and regulations of quarantine as adopted and rigidly enforced in this country and others, and theories in regard to the commencement of disease and epidemics resulting therefrom. The general appearance, as shown by post-mortem examination, was outlined and pathological appearances minutely described. He also dwelt on the total unfitness of cattle suffering from this disease for consumption as food, and

---

**Suggestion to reporters of proceedings.*—This paragraph is printed as sent to us without editing by elimination, as we usually have to do in such cases where we are absolutely ignorant of the nature of the operation—the pig may have been trephined to alter some psychological condition or it may have been gelded antiseptically—who knows? We are glad to edit hurried, rough copy, but we would like facts and data to work on.—ED.

†Prof. McEachran's mode of treatment would have been interesting information.

the liability of communicating the disease to man through the consumption of milk from dairy cows that might be afflicted. Methods of prevention by inoculation and processes for applying the different remedies were mentioned.

The subject being so important, and owing to its magnitude, it was decided to call a special meeting, which was held on Monday evening, the chair being occupied by Prof. D. McEachran, and subsequently vacated in favor of the president, Prof. Adami. The meeting was then open to its members for discussion on the above subject.

Prof. McEachran, in his opening remarks, stated the subject was a most important one to the veterinary practitioner in Canada to-day, on account of the embargo placed by England on our cattle, owing to errors in judgment of officials of the Imperial Government. He suggested the appointment of a Bacteriologist in England for the purpose of investigating bovine lung diseases with a view to the discovering by pathological and bacteriological investigations reliable means of arriving at correct dagnosis. The recently reported cases of pleuro-pneumonia contagiosa in England in cattle shipped from Canada to that country, have been investigated here, and no signs of this disease have been discovered. Portions of the lungs of two recently suspected animals have been examined microscopically by Prof. Eachran and Prof. Adami, their opinion being that it was not pleuro-pneumonia contagiosa, thus proving that this country is not affected by this disease.

The president addressed the meeting, stating that there was considerable uncertainty in dealing with pleuro-pneumonia contagiosa, and great care should be observed in diagnosing, especially for governments, thus saving trouble and expense. He advised slaughter of one animal suspected to arrive at a certainty. Prof. Adami then described minutely the appearances of the tissues and organs as presented on post-mortem examinations, describing the changes that had taken place as produced by the disease, also variations in color, size and markings, of lung tissue, etc., and the effect of diseases allied to it and their different changes as compared with this particular form of pneumonia.

Specimens were shown of genuine English pleuro pneumonia and compared with sections of what the British Veterinary Inspectors call Canadian lung, but which Prof. McEachran states would be more appropriately named transient pneumonia, as no such disease exists in Canada, and is only seen in animals travelling by railroads or an ocean journey.

After a prolonged and interesting discussion by the members, the meeting was finally adjourned for two weeks.

---

